# The P450 EcCYP89B16 broadens the metabolic landscape of herbicide resistance in barnyardgrass (*Echinochloa crus-galli)*

**DOI:** 10.1093/plphys/kiag192

**Published:** 2026-04-08

**Authors:** Praveen Khatri

**Affiliations:** Assistant Features Editor, Plant Physiology, American Society of Plant Biologists; Department of Biology, University of Toronto at Mississauga, Mississauga, ON L5L 1C6, Canada

Barnyardgrass (*Echinochloa crus-galli*) is one of the most destructive weeds in rice cultivation because its survival and adaptability can severely reduce yield and complicate crop management (Guo et al. 2017). Herbicide resistance in this species is not just a local agronomic nuisance; it shows how quickly plant populations can evolve under intense chemical selection (Powles and Yu, 2010). There are 2 broad mechanisms of herbicide resistance including target-site resistance (TSR) and non-target-site resistance (NTSR). TSR is normally caused by mutations or altered expression of herbicide target genes, which reduce herbicide binding or effectiveness. Known target genes include acetyl-CoA carboxylase (ACCase) and acetolactate synthase (ALS) (Délye et al. 2013; Gaines et al. 2020). In comparison, NTSR tends to restrict the amount of active herbicide that reaches the target site by varying routes, including reduced uptake, sequestration or enhanced metabolism (Délye 2013). Although the simplest explanation of resistance is usually the target-site mutations, they are not the whole story. The escape path of NTSR is the more evasive and alarming in many weeds, as weeds detoxify herbicides before they can get to the target sites. In this context, NTSR becomes problematic in weed management because it can spread across different classes of herbicides, making evolution of resistance mechanism harder to predict.

In this issue of *Plant Physiology*, Qi et al. (2026) provide an important new example of this logic by identifying EcCYP89B16, a cytochrome P450, as a contributor to herbicide resistance in *E. crus-galli* and show that its elevated expression is accompanied by broader metabolic and physiological changes (Qi et al. 2026).

Cytochrome P450s are especially better positioned to mediate herbicide metabolism. In land plants, P450s are one of the most diverse and versatile enzyme families, and aid in both endogenous metabolism and adaptation to the environment. They are deeply integrated into specialized metabolism, where they generate structural diversity, alter pathway flux, and assist in the generation of the extensive chemical repertoire that plants use to provide defense, communication, and adaptation (Nelson and Werck-Reichhart 2011; Hansen et al. 2021). Their catalytic flexibility is also what makes them the best candidates of xenobiotic detoxification, such as herbicide metabolism (Dimaano and Iwakami 2021).

The authors started by comparing a resistant population (R-HY) with a susceptible population (S) of *E. crus-galli*. R-HY exhibited resistance to cyhalofop-butyl, as well as cross-resistance to pinoxaden, penoxsulam, and tripyrasulfone. This immediately raised the question whether the phenotype was a result of a classical ACCase-based TSR mechanism, or of a broad metabolic form of resistance. The authors found that the phenotype was not a result of any known ACCase target-site mutations or altered ACCase expression. Rather, pretreatment with malathion, a P450 inhibitor, significantly reversed the resistance. This narrowed the phenotypic explanation to P450 metabolism-centered resistance. The authors employed transcriptome profiling in order to determine the P450 gene responsible for this phenotype. Out of hundreds of annotated P450 contigs, a single sequence of CYP89-family, *EcCYP89B16*, was significantly upregulated in the resistant population. When the authors overexpressed *EcCYP89B16* in rice using genetic transformation, the transgenic plants were more tolerant to cyhalofop-butyl, pinoxaden, and penoxsulam, but not to tripyrasulfone. These results were also complemented by HPLC-based metabolism assays in the overexpression lines and molecular docking with herbicide substrates. The combination of transcriptomics, transgenic validation, metabolism assays, and structural modeling supports the argument that *EcCYP89B16* is not simply a marker of resistance, but a legitimate herbicide-metabolizing P450.

In weeds, the best-known cases of P450-mediated herbicide resistance have been reported in CYP81 family, including the previously described *E. crus-galli* gene *CYP81A68*, which metabolizes cyhalofop-butyl (Iwakami et al. 2014; Pan et al. 2022). Other P450 family members, including *EcCYP72A385* in *E. crus-galli* and *AaCYP709C56* in *Alopecurus aequalis*, also show ability for herbicide detoxification (Zhao et al. 2022; Wang et al. 2024). Therefore, current study broadens the known enzymatic repertoire for metabolizing herbicides in resistant weeds. This study also provides the observation that metabolic resistance is not restricted to a single P450 but instead seems to represent a broadly available biochemical strategy capable of accessing a broader pool of enzymes. That is not quite unexpected, however, in terms of the specialized metabolism. P450 families are already involved in the generation of chemical diversity in plants. So, it is indeed not surprising that certain P450s can be adapted to herbicide metabolism under strong selection pressure (Délye 2013; Dimaano and Iwakami 2021).

The regulatory angle of resistance phenotype in *E. crus-galli* was also examined in this study. The authors found that the *EcCYP89B16* amino acid sequence was identical in both resistant and susceptible cultivars, suggesting that the resistance is likely caused by mis-regulation rather than biochemical differences per se. The authors subsequently detected the variation in one nearby long terminal repeat region, EcLTR3, and observed the motif differences between resistant and susceptible biotypes. The authors hypothesized that the herbicide resistance might not be due to a newly expressed enzyme activity but to the regulation of an already existing one. Such mechanisms cause the weeds to be evolutionarily tough. Herbicide resistance had already been associated with the various forms of promoter methylation, methyltransferase-mediated regulation, transcription factors, and transposable elements observed in such resistant weeds in prior research (Pan et al. 2021; Pan et al. 2022; Zhang et al. 2022; Wang et al. 2023; Wang et al. 2024; Tossolini et al. 2025). EcLTR3, in that wider context, possibly forms a component of the regulatory framework that enabled one gene of the CYP89 family to be selected as a resistance determinant due to selection pressure.

The authors also integrated transcriptomics and metabolomics to investigate what broader metabolic state accompanies *EcCYP89B16* overexpression. *EcCYP89B16* overexpression in transgenic rice was associated with 903 differentially expressed genes and 186 differentially accumulated metabolites. These changes were enriched in pathways related to glycerophospholipid metabolism, α-linolenic acid metabolism, purine metabolism, and secondary metabolite biosynthesis. The metabolome was also shifted to derivatives of amino acids, organic acids, glycerophospholipids, terpenoids, flavonoids, and alkaloids. The correlation of transcriptomic and metabolomic data highlighted several representative candidates, such as *LRR receptor-like serine/threonine-protein kinase* (LRRNT_2), *leucine-rich repeat receptor protein kinase* (MSP1), *alcohol dehydrogenase* (ADH_N), *and phospholipase D alpha 2* (PLDc_2), along with glycerophospholipids (GP), that were associated with the *EcCYP89B16* overexpression state. Together, these results provide insight into possible pathways, genes, and metabolites that can be involved in the process of EcCYP89B16-mediated herbicide detoxification.

This broader metabolic context also helps explain why the plant-growth angle is worth discussing. The authors also observed accumulation of 3-phosphoglyceric acid (3-PGA) and transcriptional shifts in genes linked to carbon fixation and Calvin-cycle-related metabolism, and, accordingly, assumed that the increases in *EcCYP89B16* expression might be attributed to the correspondence with physiological conditions that assist with the organization of stress responses and energy balance. Therefore, EcCYP89B16 may not only enhance herbicide resistance through metabolic detoxification but also be associated with broader adjustments that help balance growth and stress responses (Figure 1).

**Figure 1:**
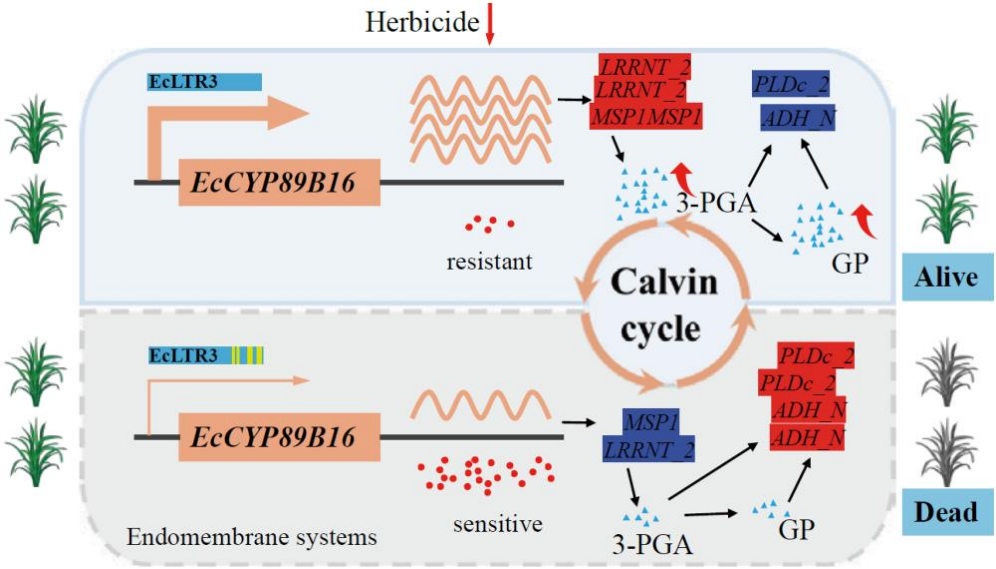
A model illustrating EcCYP89B16-mediated herbicide resistance and its coordination with the Calvin cycle. EcLTR3-driven overexpression of *EcCYP89B16* enhances the metabolic detoxification of cyhalofop-butyl, thereby conferring herbicide resistance. Concurrently, this pathway optimizes photosynthetic energy homeostasis via the Calvin cycle, thereby balancing plant growth and stress adaptation. **#** EcLTR3 (long terminal repeat); *3-PGA* (3-phosphoglyceric acid); *MSP1* (Leucine-rich repeat receptor protein kinase), *LRRNT_2* (LRR receptor-like serine/threonine-protein kinase); GP (glycerophospholipid); *ADH_N* (alcohol dehydrogenase); *PLDc_2* (phospholipase D alpha 2). Adapted from Figure 11 of Qi et al. (2026).

Collectively, for weed management, the implications of this study are clear. A resistance mechanism built on metabolic plasticity, potential regulatory variation, and broad pathway rewiring is harder to monitor and harder to suppress than a simple target-site mutation. This study supports the hypothesis that the same enzymatic toolkit enabling specialized metabolism can also be repurposed to metabolize herbicides and that regulatory modifications (which may include transposable elements) can be used to mobilize those enzymes in the face of strong selection. Future research is needed to investigate the possibility that EcLTR3 directly triggers *EcCYP89B16* overexpression, whether the associated metabolic shifts reduce fitness costs or actively support resistance, and how often similar P450-centered solutions arise in other weeds. Even now, however, the message is strong: in barnyardgrass, herbicide resistance is not merely a target-site problem. It is a systems-level metabolic adaptation, with P450 chemistry at its center.

## Data Availability

No new data were generated or analyzed in support of this research. All information needed to evaluate the study is contained within the article.
